# Ipilimumab as a Cause of Severe Pan-Colitis and Colonic Perforation

**DOI:** 10.7759/cureus.1182

**Published:** 2017-04-20

**Authors:** Raj Shah, Danielle Witt, Talal Asif, Fahad F Mir

**Affiliations:** 1 Department of Internal Medicine, University of Missouri Kansas City (UMKC); 2 Department of Gastroenterology, University of Missouri Kansas City (UMKC)

**Keywords:** ipilimumab, colon perforation

## Abstract

Ipilimumab is a human monoclonal antibody that functions as a cytotoxic T-lymphocyte-associated antigen 4 (CTLA-4) inhibitor that is used to treat malignant melanoma. Due to ipilimumab’s removal of immune regulation, specifically through the inactivation of CTLA-4, it is commonly associated with inflammatory and autoimmune events. Gastrointestinal (GI) related immune-related adverse events such as diarrhea occur in 29% of patients with 7.6% of patients specifically suffering from colitis. We describe a case of colonic perforation with ipilimumab use. Our goal is to raise awareness and alert practicing gastroenterologists of this particular adverse effect.​

A 74-year-old male patient presented to the emergency department with complaints of hematochezia, abdominal pain and decreased appetite. The patient’s past medical history included desmoplastic BRAF mutation negative melanoma with metastatic disease to the face, liver, and trigeminal nerve. He underwent his last treatment of ipilimumab three weeks prior to presentation. In total, the patient received four doses of 3 mg/kg of ipilimumab every three weeks. Since the initiation of ipilimumab, he reported diarrhea as its adverse effect, which was treated with tapering doses of prednisone one month at a time. Colonoscopy revealed mucosal ulceration and erosion in the rectum, sigmoid colon, and remaining descending colon up to the splenic flexure and cecum. After the colonoscopy, the patient became tachycardic, hypotensive and complained of sudden abdominal pain. A computed tomographic (CT) scan of the abdomen showed free intraperitoneal air. He was immediately taken to the operating room (OR) for an emergent laparotomy. In the operating room, perforations were noted at the splenic flexure and the cecum with large amounts of succus spilling from the perforations. The majority of the large bowel appeared cyanotic and dusky; consequently, a sub-total colectomy with terminal ileostomy was performed. After the procedure, the patient was started on antibiotics for severe peritonitis and admitted to the intensive care unit (ICU) with septic shock. His clinical status continued to deteriorate due to acute respiratory failure, nosocomial pneumonia, severe protein calorie malnutrition and coagulopathy from disseminated intravascular coagulation (DIC). The patient did not recover from his illness and died a few days later.

It is imperative that physicians caring for patients receiving treatment with CTLA-4 inhibitors frequently monitor for and promptly treat possible immune-related adverse effects. For patients with ipilimumab-related colitis, prompt identification of symptoms and early treatment with steroids are crucial in preventing harmful or possibly fatal immune-related adverse events. Gastroenterologists should be wary of this adverse side effect in this high-risk population when performing colonoscopy and take necessary precautions.

## Introduction

Our case report describes a case of hematochezia in a patient who recently completed treatment with ipilimumab for diffuse metastatic melanoma. The patient was found to have severe colitis complicated by bowel perforation after colonoscopy. Gastrointestinal (GI) toxicity, including diarrhea and colitis, is a known adverse effect of ipilimumab. Due to ipilimumab’s removal of immune regulation, specifically through the inactivation of cytotoxic T-lymphocyte-associated antigen 4 (CTLA-4), it is commonly associated with inflammatory and autoimmune events. It is imperative that physicians caring for patients receiving treatment with CTLA-4 inhibitors frequently monitor for and promptly treat possible immune-related adverse effects. This case report aims to raise awareness of these potentially devastating side effects and help guide practicing physicians.

## Case presentation

A 74-year-old male patient presented to the emergency department with complaints of hematochezia. The patient reported bright red blood coating his stool since that afternoon. Additionally, he complained of mild abdominal pain and decreased appetite. He also mentioned diarrhea intermittently throughout ipilimumab infusions for his malignant melanoma. Prednisone was administered at the onset of each of these diarrheal episodes, and he responded to treatment consistently. His last cycle of ipilimumab was three weeks prior to presentation, and he had not developed diarrhea at that time. In total, the patient had received 3 mg/kg of ipilimumab every three weeks for a total of four doses. The patient denied use of non-steroidal anti-inflammatory drugs (NSAIDS) and any history of radiation to the bowel. He also denied any other symptoms such as hematemesis or melena. There were no sick contacts at home.

Our patient’s past medical history included metastatic melanoma with metastatic disease to the face, liver, and trigeminal nerve for which he received stereotactic radiation. Pathological and cytological examination of the primary and metastatic sites of melanoma revealed desmoplastic BRAF mutation negative melanoma. The past medical history was also significant for chronic constipation, neuralgia, and hypertension. The patient’s remote surgical history included a transverse and descending colectomy for a mass, which was later reported to be a colonic lipoma on histopathology, and partial hepatectomy for metastatic melanoma. He did not have any history of peripheral arterial disease, cerebrovascular accident or coronary artery disease to suggest an atherosclerotic process. Computed tomographic (CT) scan of the abdomen obtained six months prior had shown patent intra-abdominal vascular structures.

On presentation, blood pressure was 124/85, pulse 85/minute, temperature 98.6°F, respiratory rate 18/minute and oxygen saturation 99%. On physical exam, the patient appeared cachectic but in no acute distress. He had diffuse abdominal tenderness to deep palpation.

Initial labs, especially the basic metabolic panel, showed hypokalemia (3.3 meq/L), hyponatremia (128 meq/L), and mild metabolic alkalosis (bicarbonate 28 meq/L). Renal functions and liver function tests were within normal limits. There was no leukocytosis. Hemoglobin (Hgb) at presentation was 9.7 g/dl, which decreased to 7.8 g/dl and stabilized. He was typed and screened, started on intravenous fluids, and his electrolytes were replaced. Stool studies were ordered to identify any infectious cause of the diarrhea and hematochezia. However, samples could not be obtained as he did not have a bowel movement.

**Figure 1:**
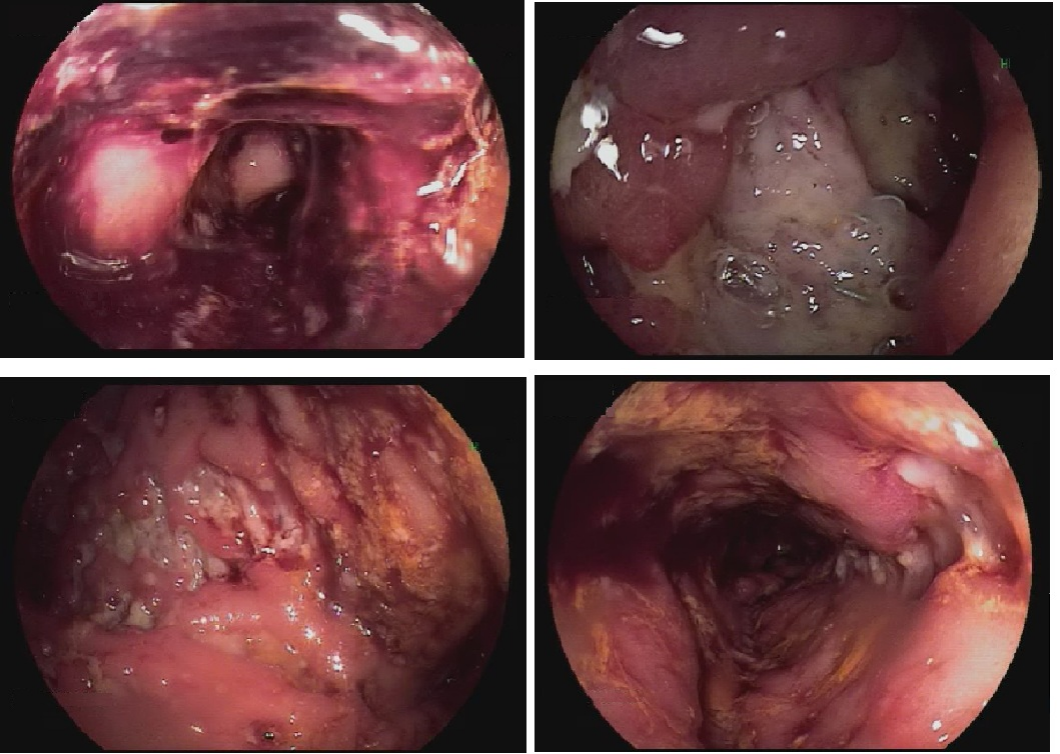
Ulcerations in the sigmoid colon, descending colon upto splenic flexure and cecum.

He was started on empiric 1 mg/kg methylprednisolone for suspected ipilimumab-induced colitis. Due to concern for acute gastrointestinal (GI) bleed and a decreasing trend in the patients Hgb, the patient was started on intravenous pantoprazole and underwent a colonoscopy and esophagogastroduodenoscopy (EGD) to rule out a brisk upper GI bleed. No abdominal imaging was obtained at this time. The EGD was unremarkable. Colonoscopy revealed mucosal ulceration and erosion in the rectum, sigmoid colon, remaining descending colon up to the splenic flexure and cecum, consistent with severe colitis (Figure 1). The ascending colon was spared. Colocolonic anastomosis at the sigmoid colon was seen and was intact.

**Figure 2:**
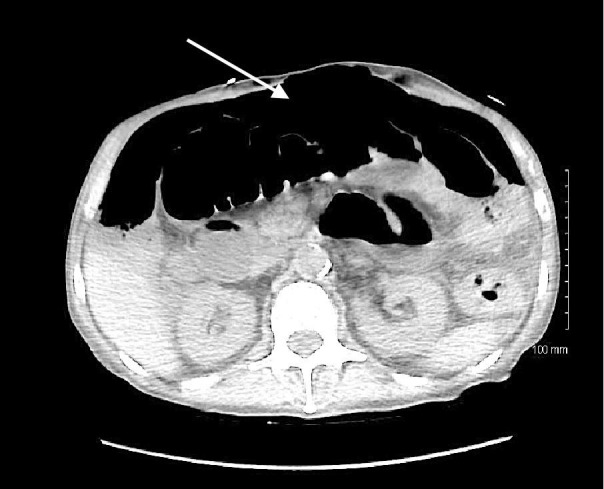
Computed tomography (CT) abdomen demonstrating large free intraperitoneal air (arrow) consistent with bowel perforation.

After colonoscopy, the patient complained of sudden worsening of abdominal pain, became hypotensive and tachycardic. A CT scan of the abdomen was obtained emergently that revealed large free intraperitoneal air (Figure 2).

There was mild diffuse colonic wall thickening. The patient was taken to the operating room (OR) for an emergent laparotomy. In the OR, perforations were noted at the splenic flexure and the cecum with large amounts of succus spilling from the perforations. The majority of the large bowel appeared cyanotic and dusky. Consequently, a sub-total colectomy with terminal ileostomy was performed. The patient was admitted to the intensive care unit and started on broad spectrum intravenous antibiotics for secondary peritonitis and septic shock. However, his clinical status continued to deteriorate including acute respiratory failure, nosocomial pneumonia, severe protein calorie malnutrition and coagulopathy from disseminated intravascular coagulation. After the acute deterioration, his family did not want further intervention and opted for comfort care. He died a few days later.

Severe colitis resulting from ipilimumab use was diagnosed based on pathology from surgical specimens, distribution of colitis on endoscopy and no other predisposing factors. Histologic analysis of the specimen showed severe acute inflammation. Distribution of the colitis did not follow any vascular pattern, hence providing even more evidence to differentiate it from ischemic colitis. The severe colitis predisposed to iatrogenic perforation and significant morbidity in this case.

## Discussion

Ipilimumab is an human monoclonal antibody that functions as a cytotoxic T-lymphocyte-associated antigen 4 (CTLA-4) inhibitor used for the treatment of metastatic melanoma [1]. CTLA-4 prevents T-cell activation by competing for binding of surface proteins on antigen presenting cells that would normally cause T-cell activation. Through this action, CTLA-4 functions to limit immune responses and reduce autoimmune reactions [2]. Therefore, ipilimumab, a CTLA-4 antibody, functions as an immune checkpoint inhibitor that prevents the inactivation of T-cells whose activity may be helpful in tumor-related immune responses. Through the removal of immune inhibition, ipilimumab results in immune-related adverse effects (irAE). The most well-known adverse effects of ipilimumab involve gastrointestinal, dermatologic, and endocrine toxicities [3]. According to a phase 3 study, irAEs occurred in as many as 80% of patients receiving ipilimumab monotherapy for metastatic melanoma [1]. GI-related irAEs such as diarrhea occurred in 29% of patients with 7.6% of patients specifically suffering from colitis [1].

Although ipilimumab is known to cause some level of dysregulation of colonic mucosal immunity secondary to inflammation caused by immune activation, the exact mechanism of ipilimumab’s GI toxicity is not fully understood. It appears to involve a mechanism independent of other inflammatory conditions of the bowel. Upon examination of colitis related to ipilimumab, there is histological evidence of mucosal inflammation and an increase in neutrophil-derived fecal calprotectin like in other inflammatory bowel diseases (IBD) [4]. However, in classic inflammatory bowel diseases such as Crohn’s disease (CD) and ulcerative colitis (UC) specific antibody patterns to enteric flora are noted. The titer pattern noted in patients suffering from colitis secondary to ipilimumab treatment was distinct to the patterns observed in CD and UC [4]. Histologically, ipilimumab-related colitis more closely resembles UC with a pattern of more distal involvement; however, the classic chronic histological findings of ulcerations and granulomas, with UC and CD, respectively, are not present in ipilimumab-related colitis. Histological findings in ipilimumab-related colitis can range from normal architecture to severe acute inflammation [5]. Although studies have sought to identify indicators of patients that will be affected by ipilimumab-related colitis, no specific biomarkers have been identified [4].

Colonoscopy can be useful in confirmation of a suspected cause of colitis and also in diagnosing the cause of chronic diarrhea. Not only does colonoscopy allow for direct visualization of colonic integrity, it provides the opportunity to obtain biopsies for histological examination. In a study that examined 168 patients with non-human immunodeficiency virus (HIV) related chronic diarrhea, a histological diagnosis was made in 52 patients (32%) with 14 of those patients having significant histological disease with a normal-appearing bowel [6]. In our patient, the likely cause of colitis seen on colonoscopy and CT abdomen was ipilimumab induced. However, due to the chronicity of symptoms and previous treatment with prednisone without resolution, colonoscopy to confirm the diagnosis was imperative.

Current studies indicate that resolution of irAEs such as colitis depends on prompt reporting of symptoms, treatment with intravenous (IV) or oral high dose steroids, and temporary or permanent discontinuation of ipilimumab [7]. Steroids are started at 0.5-1 mg/kg/day and tapered over a one-month period. Most patients respond to treatment with steroids. Refractory cases (no improvement after three days of steroids) are treated with infliximab. Mycophenolate is the next option in infliximab-resistant cases. In a retrospective study of 836 patients who received ipilimumab through clinical trials, faster resolution was noted if steroids were initiated within five days of onset [8]. The most common time for any irAE to occur is the induction period of the drug (within 12 weeks of initial dosing). These data emphasize the importance of the physicians' diligence in monitoring their patients for adverse effects throughout treatment [1]. A few case reports have indicated that ipilimumab has the potential to cause colonic perforation [9-10]. Gastroenterologists should be aware and wary of this adverse effect in this high-risk population when performing colonoscopy and take necessary precautions.

## Conclusions

Colonoscopy should be performed with great caution in patients with diarrhea who are receiving ipilimumab treatment due to the risk of iatrogenic perforation. Case reports like these carry great value as they highlight and raise awareness of devastating consequences of treatment with ipilimumab so that prompt preventive measures can be taken.
